# A Rare Case of Ovarian Dermoid Torsion in the Third Trimester: Clinical Challenges and Surgical Management

**DOI:** 10.7759/cureus.95643

**Published:** 2025-10-29

**Authors:** Raisham Saleem, Poornima Sathyendran, Tasbieh Askalany, Roxana Mo

**Affiliations:** 1 Obstetrics and Gynaecology, Mid and South Essex NHS Foundations Trust, Southend-on-Sea, GBR; 2 Obstetrics and Gynaecology, Luton and Dunstable University Hospital, Luton, GBR; 3 Obstetrics and Gynaecology, Southend University Hospital, Southend-on-Sea, GBR

**Keywords:** acute abdomen in pregnancy, adnexal torsion, caesarean section, gynecological emergency, maternal-fetal outcomes, ovarian cystectomy, ovarian dermoid cyst, pregnancy complications, third trimester pregnancy

## Abstract

Adnexal torsion is an uncommon but serious complication of pregnancy, particularly rare in the third trimester, where it poses challenges for both diagnosis and management. We report the case of a 25-year-old primigravida at 36+3 weeks of gestation with a known right ovarian dermoid cyst who presented with acute abdominal and back pain. She had experienced multiple previous admissions for abdominal pain that were managed conservatively. Initial investigations, including ultrasound and MRI, confirmed the presence of an 8 cm complex right ovarian mass but did not conclusively demonstrate torsion. Despite conservative management and antibiotic therapy for suspected sepsis, her pain worsened, and laboratory results showed raised inflammatory markers. Owing to persistent symptoms and clinical deterioration, an emergency caesarean section with concurrent surgical management was undertaken. Intra-operatively, the right ovary was found to be twisted four times, and a cystectomy with partial oophorectomy was performed along with delivery of a healthy female infant. Postoperative recovery was favourable, and histopathology confirmed a dermoid cyst. This case highlights the diagnostic uncertainty of adnexal torsion in pregnancy, where overlapping symptoms can obscure recognition. It underscores the importance of maintaining a high index of suspicion, early multidisciplinary involvement, and timely surgical intervention to optimize maternal and neonatal outcomes.

## Introduction

Adnexal masses are a relatively frequent incidental finding during routine antenatal ultrasound examinations. In most cases, these lesions are benign or functional in nature and either remain clinically insignificant throughout pregnancy or regress spontaneously by the second trimester. Lesions measuring more than 5 cm, or those with atypical imaging features, warrant closer surveillance and may occasionally require surgical management during gestation. Malignant ovarian tumors, however, are exceedingly uncommon in pregnancy. It is estimated that nearly one-quarter of adnexal torsion cases occur in association with pregnancy.

The reported incidence of torsion among adnexal masses during pregnancy ranges from 2% to 16%, with the majority of cases identified in the first trimester. After 20 weeks of gestation, the likelihood drops below 5%, and torsion in the third trimester is particularly rare, making the diagnosis challenging.

Accurate diagnosis relies on careful clinical assessment supported by imaging. Ultrasound is the first-line modality, while MRI is valuable in excluding other differential diagnoses. Laboratory investigations are generally unhelpful in confirming torsion. Surgical management remains the cornerstone of treatment, and the choice of procedure is influenced by gestational age and the level of suspicion for malignancy. In late pregnancy, surgical intervention may be combined with delivery by caesarean section when indicated.

Here, we describe a rare case of a 25-year-old woman in her third trimester who presented with ovarian torsion due to a large dermoid cyst. She underwent a caesarean section with detorsion, right ovarian cystectomy, and partial oophorectomy, resulting in a favorable maternal and neonatal outcome.

## Case presentation

A 25-year-old primigravida (G2P0+1) at 36+3 weeks of gestation presented to the maternity triage department with acute abdominal and back pain. She had a known 8 cm right ovarian dermoid cyst and had three previous admissions for abdominal pain, which were managed conservatively with analgesics. The patient had a normal BMI and no known drug allergies.

Initial investigations, including ultrasound, showed a right ovarian terato-dermoid cyst measuring 4.6 × 7 × 8.3 cm. MRI revealed a mixed solid-cystic lesion consistent with a right ovarian dermoid without signs of torsion or appendicitis. A routine growth scan at 35+2 weeks showed normal fetal development, and she had a plan for induction of labour at 38 weeks. Despite conservative management, her pain worsened over the course of one week, and she also experienced nausea and vomiting.

Blood tests showed elevated C-reactive protein (CRP) and white blood cell (WBC). She was admitted to the antenatal ward and started on intravenous antibiotics for a suspected urinary tract infection. Her abdomen was soft, with generalized tenderness more pronounced in the right iliac fossa. No contractions were felt, and the cervical os was closed on speculum examination. Cardiotocography (CTG) was reassuring. Urgent MRI showed minimal reactive fluid around the cyst, with torsion not completely excluded.

**Figure 1:**
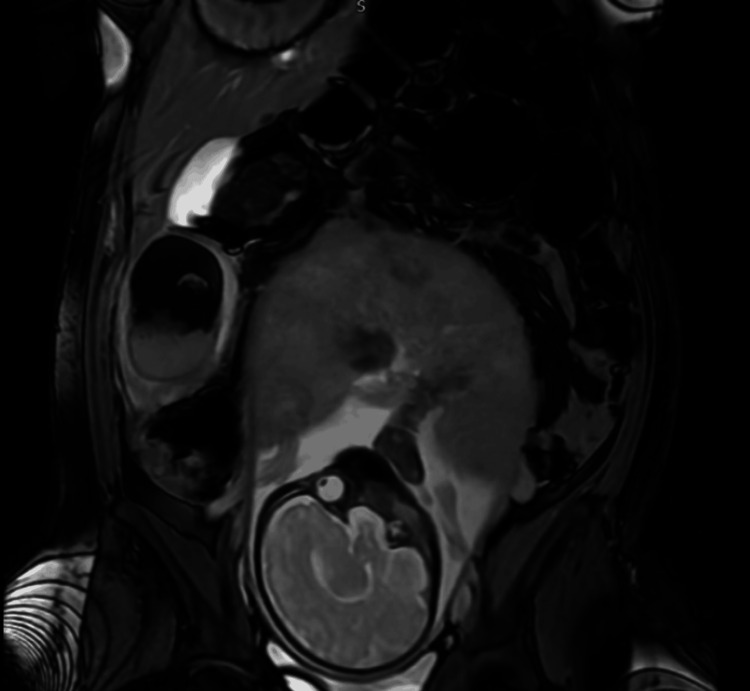
MRI showing the gravid uterus with the fetus and a right adnexal mass (dermoid cyst)

**Figure 2:**
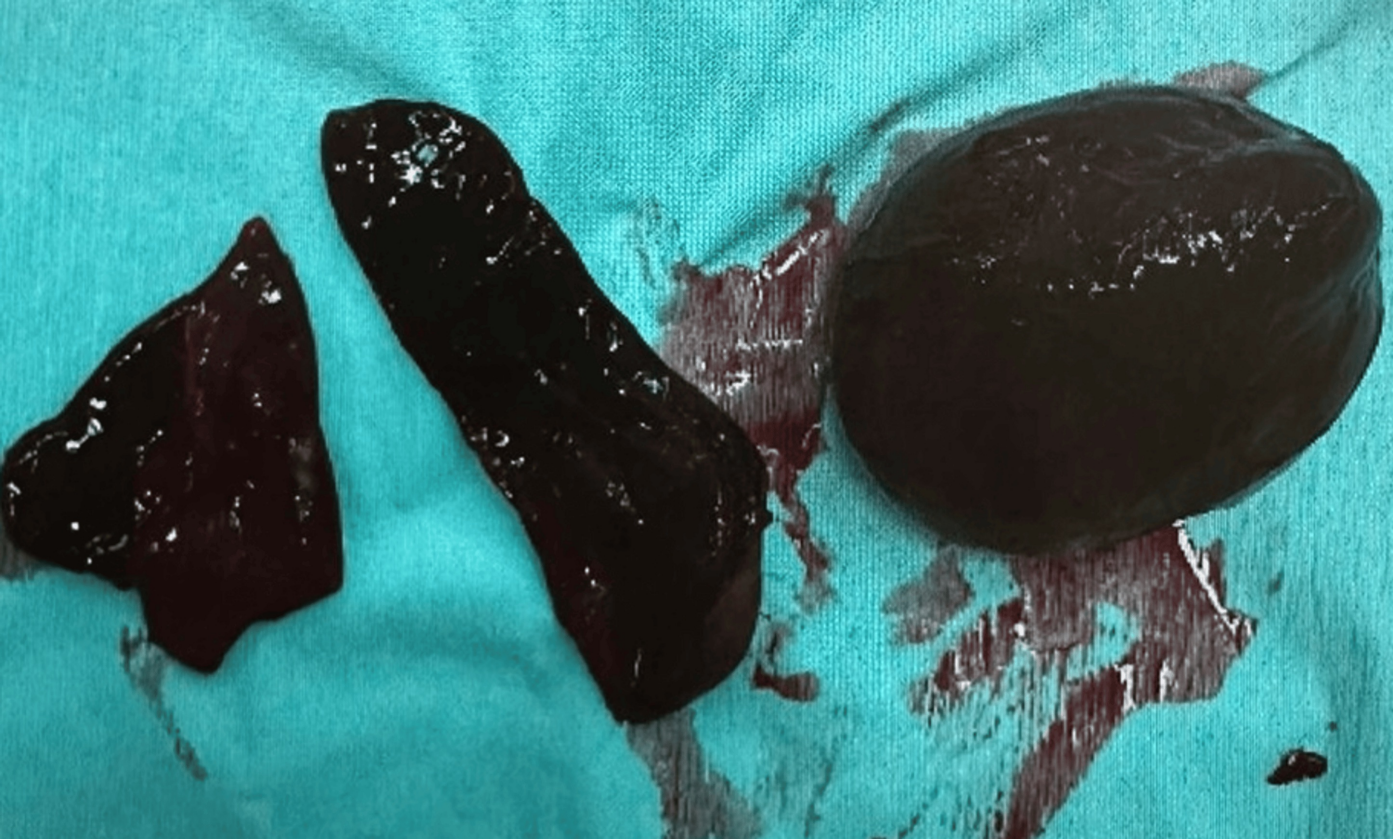
Twisted adnexa demonstrating multiple turns of the vascular pedicle leading to ovarian ischemia

**Figure 3:**
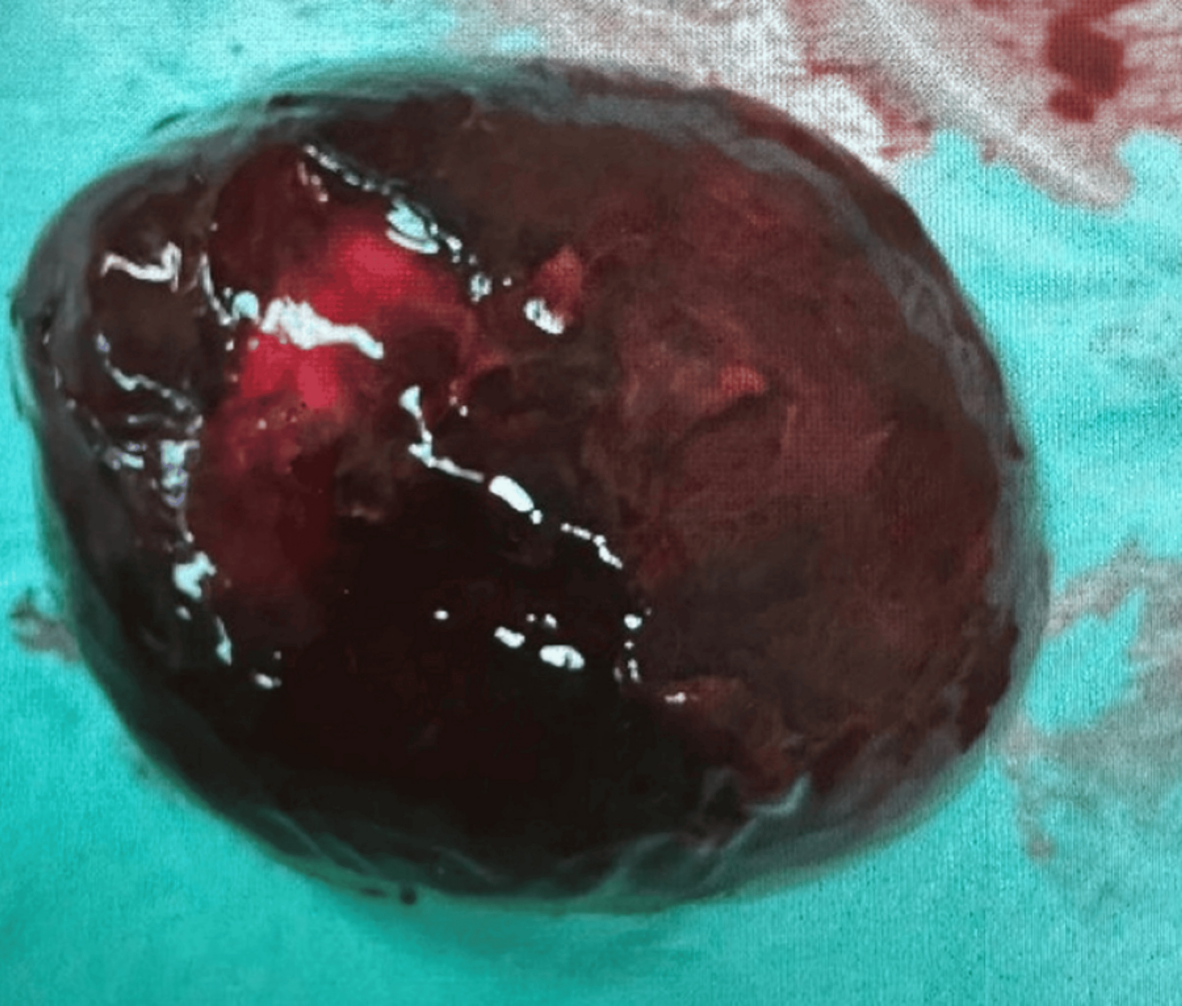
Gangrenous ovary showing darkened, necrotic tissue with loss of the normal ovarian architecture

The case was discussed with the on-call obstetrician and other senior members of the team. Based on her persistent pain and suspicion of sepsis, caesarean section and detorsion of the right ovary, along with right ovarian cystectomy, were performed at 36+4 weeks. Intra-operatively, the ovary was found to be twisted four times on its axis, which partially regained colour after detorsion. The patient experienced significant blood loss but was stabilized with medications and transfusions. She delivered a healthy female baby weighing 2.66 kg. Postoperatively, she received antibiotics and enoxaparin. Histology confirmed the diagnosis of a dermoid cyst. Table 1 presents the laboratory results. Figure 1 presents the MRI of the patient, Figure 2 shows the twisted adnexa demonstrating multiple turns of the vascular pedicle, and Figure 3 shows the gangrenous ovary.

**Table 1:** Laboratory results of the patient on presentation

Parameter	Patient Result	Reference Range	Units
Hemoglobin	116	115–165	g/L
White Cell Count	15	4.0–11.0	×10⁹/L
Platelet Count	446	150–400	×10⁹/L
C-Reactive Protein	275	<5	mg/L

## Discussion

Adnexal masses are increasingly identified during pregnancy due to the widespread use of antenatal ultrasound. Most are benign, such as corpus luteal, follicular, or hemorrhagic cysts, and often resolve spontaneously without intervention. Ovarian torsion, although rare, carries significant maternal and fetal morbidity if not promptly diagnosed and treated [1,2].

Current Royal College of Obstetricians and Gynaecologists (RCOG) guidelines recommend conservative management for benign cysts smaller than 5 cm in premenopausal women, with close monitoring for larger or persistent masses during pregnancy [2]. Persistent cysts in the second or third trimester may require surgical intervention, particularly if the mass increases in size or causes complications. Adnexal torsion occurs due to partial or complete rotation of the adnexa around its vascular pedicle, leading to venous and arterial obstruction, ischemia, and potential necrosis [3]. Typical sonographic signs include ovarian enlargement, peripheral follicles, stromal edema, adnexal cysts, and free pelvic fluid, though these may be less apparent during pregnancy [4,5]. MRI serves as a valuable second-line modality, aiding in differential diagnosis while avoiding ionizing radiation [6].

Surgical intervention is the definitive treatment for torsion and may be performed via laparoscopy or laparotomy. The approach depends on gestational age, cyst size, suspicion of malignancy, prior surgeries, and surgical expertise. Laparoscopy is generally preferred in early pregnancy (<12 weeks), while laparotomy may be safer in the second or third trimester. Conservative procedures, such as detorsion with cystectomy or fenestration, can preserve ovarian function, with studies showing 91-100% restoration of ovarian tissue even in cases of ischemic-appearing ovaries. Both cystectomy and oophorectomy have been described during pregnancy, with no clear superiority for maternal or fetal outcomes [3,5].

Our case illustrates several practical and clinical challenges. Diagnostic uncertainty arose due to atypical presentation and overlapping symptoms, compounded by fatigue during overnight deterioration. Gestational considerations, including timing of corticosteroid administration and fetal maturity, further complicated decision-making. Prompt multidisciplinary input, situational awareness, and early surgical intervention enabled a favorable outcome. The patient underwent ovarian cystectomy with partial oophorectomy at the time of caesarean section, recovering fully within three days, while the neonate was delivered in good condition despite maternal sepsis. This case underscores the importance of listening to subjective symptoms, requesting second opinions in atypical presentations, and ensuring MDT involvement to optimize maternal and fetal outcomes.

## Conclusions

Adnexal torsion in late pregnancy is rare but requires timely recognition and surgical intervention to prevent maternal and fetal morbidity. This case highlights the importance of maintaining a high index of suspicion, especially when symptoms persist despite conservative management. Multidisciplinary involvement and prompt surgical decision-making ensured positive outcomes for both the mother and the baby.

Human factors played a significant role in this case. Respecting patient-reported symptoms, involving senior clinicians early, and effective teamwork were crucial in overcoming these challenges. Integrating human factors into clinical practice can improve decision-making and enhance maternal-fetal safety in obstetric emergencies.
